# Experimental investigation of drag loss behavior of dip-lubricated wet clutches for building a data-driven prediction model

**DOI:** 10.1038/s41598-024-59488-4

**Published:** 2024-04-22

**Authors:** Lukas Pointner-Gabriel, Max Menzel, Katharina Voelkel, Thomas Schneider, Karsten Stahl

**Affiliations:** School of Engineering and Design, Department of Mechanical Engineering, Gear Research Center (FZG), Technical University of Munich, Boltzmannstrasse 15, 85748 Garching near Munich, Germany

**Keywords:** Fluid dynamics, Mechanical engineering

## Abstract

Fundamental knowledge of wet clutches’ drag loss behavior is essential for designing low-loss clutch systems. In contrast to the widely investigated injection lubrication, more comprehensive knowledge is needed on the drag loss behavior of dip-lubricated wet clutches. In the development phase, data-driven models allow drag loss predictions with low computational effort and, at the same time, sufficient accuracy. Therefore, this study aimed to deepen and expand knowledge of the drag loss behavior of dip-lubricated wet clutches based on experimental investigations. Moreover, the investigations were designed and conducted so that the generated data and findings can be used in further research for building data-driven prediction models. The investigations were conducted on two clutch systems from automotive and industrial applications. The practice-relevant parameters of clearance, oil level, oil viscosity, and plate shape were investigated based on a mixed-level full factorial design. The evaluation shows that a reduction in drag loss can be achieved primarily by increasing the clearance, reducing the oil viscosity, and choosing waved plates. The obtained drag loss behavior can be traced back to the form of oil displacement from the gaps. The displacement process, in turn, is influenced by the operating and geometry parameters. Although the flow in the gaps develops differently for dip and injection lubrication over differential speed, the study shows comparable integral effects of the influencing parameters for both types of lubrication. The generated datasets contain the investigated parameters as features and characteristic drag loss values as targets. The findings can support the selection and configuration of the machine learning algorithm and the validation of the trained models. The described procedure can serve as a template for generating and analyzing datasets for data-driven modeling of wet clutches’ drag losses.

## Introduction

Wet-running multi-plate clutches (hereinafter referred to simply as wet clutches) represent key components in modern drive technology. They can be engaged under differential speed, and the torque can be flexibly adjusted even during operation. However, in the disengaged state and under differential speed, hydrodynamic drag losses occur, mainly due to the shearing of the oil in the gaps. These losses can account for a considerable percentage of the total losses in a drivetrain^1^. Fundamental knowledge of the drag loss behavior and calculation models are essential for designing low-loss clutch systems. Hence, wet clutches have been the subject of ongoing research since the 1970s^2,3^ to date^4,5^. The latest research covers experimental investigations of the integral drag loss behavior^6,7^, in-depth investigations^4,8,9^ of the oil flow and drag torque generation in the gaps, and applying advanced technologies^10,11^. Additionally, research is done on analytical^12,13^, numerical^5,9,14,15^, and data-driven^5,16^ models to calculate the drag losses.

The type of lubrication applied mainly depends on the system requirements. In the case of injection lubrication, the oil is actively injected from the inside, offering the advantage of controlling the cooling performance through variable flow rate adjustment. In contrast, in the case of dip lubrication, the plates are permanently immersed in an oil sump of constant volume, generally resulting in a lower cooling performance but a less complex lubrication system.

**Figure 1:**
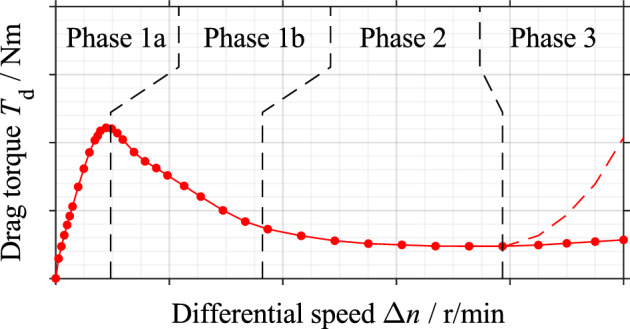
Characteristic drag torque curve of a wet clutch and its classification ^16^.

Usually, the drag loss behavior is described by drag torque over differential speed and can be classified into three characteristic phases (see Fig. 1). With increasing differential speed, the drag torque first increases to a maximum value (Phase 1a), then drops to a low level (Phase 1b), remains at an almost constant value (Phase 2), and may re-increase abruptly at high speeds (Phase 3).

Extensive research studies focusing on injection-lubricated wet clutches found that numerous design and operating parameters influence the drag loss behavior. Today, it is known that drag losses can be reduced, e.g., by reducing facing area^17^, oil viscosity^3,18–20^, or injection flow rate^3,17–19^, as well as by increasing clearance^3,18–20^, using waved plates^3,17,18^, and optimizing groove design^17,19–22^.

Base studies found that the drag torque curve shows identical characteristics for dip and injection lubrication^4,7^. However, the flow development in the gap differs between the two lubrication types^4^. Independent of the lubrication type, the gaps are filled with oil at low differential speeds (Phase 1a), meaning a single-phase flow is present. In the case of injection lubrication, air enters the gaps from the outside^23^ when the conveying rate of the clutch exceeds the flow rate supplied, resulting in a two-phase flow (Phase 1b). The fraction of air increases with increasing differential speed (Phase 2)^24^. In contrast, during dip lubrication, the oil is continuously displaced from the gaps due to increasing centrifugal forces starting from the inside^4^. The sudden re-increase in drag torque (Phase 3) can be traced back to speed-induced phenomena^25,26^.

The vast majority of the investigations were conducted for injection-lubricated wet clutches. However, to the best of the authors’ knowledge, comparatively few investigations^7,18,27^ have been conducted on the drag loss behavior of dip-lubricated wet clutches. Wet clutches of automotive limited-speed differentials or industrial transmissions, e.g., are typically dip-lubricated. It is known that the oil level has to be kept low for minimum drag losses^7,18,27^. In a preliminary study ^7^ for the investigations presented in this paper, the influencing parameters of clearance, oil viscosity, oil level, number of gaps, and groove design were identified based on a two-level full factorial test design but not discussed in detail. Thus, there is a lack of current research on dip-lubricated wet clutches since the effects of further practically relevant design and operating parameters have yet to be investigated and discussed in detail. Also, potential non-linear effects of influencing parameters on the drag loss behavior have yet to be investigated.

CFD models^28–35^ and analytical models^35–42^ are available to determine the drag losses of wet clutches. CFD models are considered highly accurate but computing-intensive, whereas analytical calculation models allow a rough but quick estimation^16^. The CFD calculation of the drag torque at a specific differential can last up to 8 h on a workstation with 32 cores^28^. Analytical models are based on the Navier–Stokes equations and are based on several assumptions^20^. The simplified modeling of the complex two-phase flow partly leads to a considerable reduction in accuracy^20^. Further, the above-cited CFD and analytical models are limited to injection lubrication. In contrast, data-driven modeling enables building prediction models for any complex physical effects and lubrication type^16^. On top of that, data-driven models enable the drag losses to be predicted with low computational effort and, at the same time, sufficient accuracy^16^. Drag torque measurements from systematic investigations on the influence of various geometry and operating parameters on drag loss behavior are needed to build the prediction models^16^. However, no datasets are publicly available. Thus, there is a lack of research data for building advantageous data-driven prediction models supporting the development of low-loss clutch systems.

The main goal of the study was to deepen and expand knowledge of the effects of practically relevant design and operating parameters (i.e., clearance, oil level, oil viscosity, groove angle, and plate shape) on the drag loss behavior of dip-lubricated wet clutches by conducting systematic (i.e., mixed-level full factorial design) experimental investigations (i.e., test rig testing) on two clutch systems of different application fields (i.e., automotive and industrial). The investigations were designed and conducted so that the generated research data and findings can be used in further research for building data-driven prediction models. Additionally, this paper represents a general template for data generation and analysis of wet clutches’ drag losses. The theoretical results of the study provide essential knowledge for developing low-loss clutch systems.

## Materials and methods

### Clutch systems and oils

**Figure 2:**
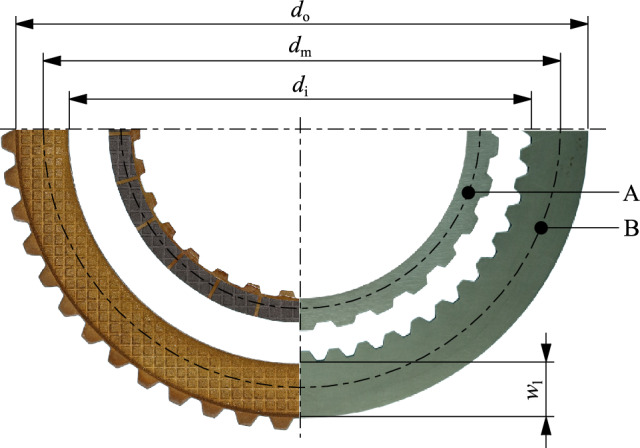
Photographs of the friction (left) and separator plates (right) of Clutch Systems A and B.

Two clutch systems from different applications were selected for the investigations. The clutch systems consisting of the plates and carriers are shown graphically in Supplementary Fig. 2. Photographs of the plates can be seen in Fig. 2. The dimensions and other essential characteristics of the clutch systems are listed in .

Table 1. Clutch System A represents an automotive application, and Clutch System B represents an industrial application. The plates were used in new condition.

**Table 1:** Dimensions and characteristics of investigated clutch systems.

	Clutch System A	Clutch System B
Application	Automotive	Industrial
*d*_i_, *d*_o_, *d*_m_, *w*_l_/mm	165, 187.5, 176.25, 11.25	198, 243, 220.5, 22.5
*N*/-	8	8
Friction lining	Paper-based	Sinter-metallic
Groove design	Radial + waffle	Waffle

The groove design of the friction plates of Clutch System A represents a superposition of a radial groove design and a waffle groove design (see Fig. 2 and Fig. 5). The friction plates each have 24 radial grooves. There are two variants of friction plates with different groove angles. A groove angle of *ϕ* = 30° (see Fig. 5 (a)) describes an inclination of the grooves to the radial line. This allows a conveying or blocking flow behavior to be realized depending on the direction of rotation. A groove angle of *ϕ* = 0° (see Fig. 5 (b)) describes purely radial grooves. In this case, the flow and drag loss behavior is theoretically independent of the direction of rotation. The separator plates of Clutch System B can be planar or waved. Waved plates improve the separation and, consequently, drag loss behavior. Constraint forces are released during the disengagement of the clutch, supporting the plates to distribute. Four waves of 0.35 mm height define the waviness of the separator plates. The separator plates are paired with planar friction plates. Both clutch systems were operated with application-specific oils. Relevant characteristics of the oils used are listed in Table 2.

**Table 2:** Physical characteristics of oils used.

Clutch System	*ρ* (15 °C)/kg/m^3^	*ν* (40 °C)/mm^2^/s	*ν* (100 °C)/mm^2^/s	*µ* (40 °C)/mPa∙s	*µ* (55 °C)/mPa∙s	*µ* (100 °C)/mPa∙s
A	840	26.8	5.6	22.1*	13.3*	4.4*
B	878	93.3	14.4	80.4*	44.2*	11.9*

Note: *values calculated according to DIN 51563^43^ and DIN 51757^44^.

### Test set-up

The investigations were conducted on the LK-4 drag torque test rig (Gear Research Center (FZG), Technical University of Munich). Supplementary Fig. 3 graphically shows the set-up of the test rig. The set-up and function principle of the test rig are described in detail in Ref.^16^. The LK-4 drag torque test rig can be used to determine the drag torque of a range of clutch sizes and configurations under different operating conditions.

**Figure 3:**
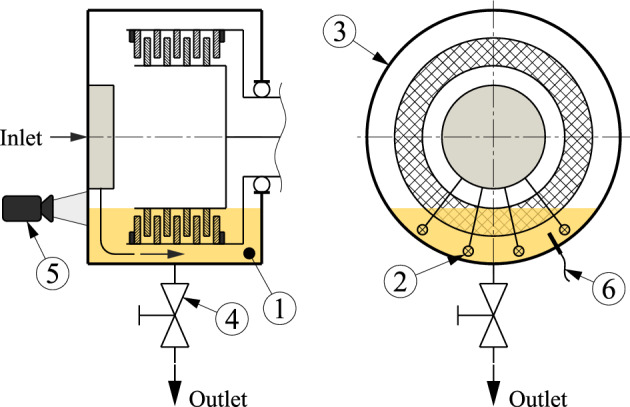
Schematic side (left) and front view (right) of the test set-up, based on Ref. ^7^.

Figure 3 shows the test set-up schematically. The housing is initially filled to a defined oil level in real applications. Due to the changing flow conditions and the permanent energy input, the oil level and sump temperature vary during operation^4^. The changing conditions, in turn, affect the resulting drag torque^4^. To ensure constant operating conditions, tempered oil was continuously injected into the oil sump (1) at four points (2). The resulting oil circulation in the housing (3) helps ensure a homogeneous temperature distribution. The flow rate fed in was drained via a valve (4). Since the oil input and drain are not connected via the test rig control system, the oil level was permanently monitored with a camera system (5) enabling the test rig operator to instantly adjust the oil level in case of variations. A thermocouple (6) was installed to monitor the oil temperature.

### Test conditions and procedure

Due to practical relevance, the investigations focused on brake operation mode with a rotating inner carrier. During this operation mode, the outer carrier is stationary. In this case, the differential speed corresponds to the speed of the inner carrier. Further, the investigations focused on Phases 1 and 2. No elements maintaining equal clearances were used, meaning the plates could move within the set total clearance. On the LK-4 drag torque test rig, the total clearance is set using a spacer ring of a defined width (see part 10 in Supplementary Fig. 3). The thickness of the clutch pack was determined according to Ref.^27^ to set the total clearance. Supplementary Fig. 4 shows the set-up used to measure the clutch pack thickness. The unmounted clutch packs were loaded with a mass of 5 kg during the measurement to ensure a repeatable state. The waved separator plates of Clutch System B were not significantly deformed under load due to their high axial stiffness. The nominal clearance per gap was used to characterize the clearance configuration. The nominal clearance occurs in the case of an equidistant distribution of the total clearance to the gaps. When using waved separator plates, the circumferential clearance varies sinusoidally. In this case, the clearance represents a superposition of the set nominal clearance and the sinusoidal waviness of the plate. The waved separator plates were not aligned in a specific manner but were installed identically in all test runs.

The tests were performed according to the procedure described in Ref.^16^. Here, the drag torque is measured stepwise for defined differential speeds. The stepwise procedure enables the determination of the steady drag torque compared to a speed ramp with continuous angular acceleration. First, the inner carrier is accelerated to the maximum speed. The initial speed ramp-up to the maximum speed supports redistribution of the plates in the set total clearance, especially after a change in the clutch set-up. This is confirmed by the high repeatability of the measurements (see Fig. 11). The starting speed was determined for each clutch system in a screening test and was set to 2000 r/min accordingly. The inner carrier speed is then stepwise reduced to a standstill. The step duration and size depend on the measurement phase and range from 30 to 120 s and 25 to 150 r/min, respectively. The clutch components were tempered to test temperature for at least five minutes at low differential speed before the test started.

### Test design

The parameters of clearance, oil level, and oil viscosity were investigated for both clutch systems. Additionally, the angle of the radial grooves was investigated for Clutch System A. Further, the shape of the separator plates was studied for Clutch System B.

In the experimental investigation of the drag losses, most of the test parameters cannot be arbitrarily varied. This decisively affects the choice of test design. Theoretically, clearance, groove angle, and oil level can be set to any value within practical limits. However, the clearance variation is limited to specific values due to the adjustment via corresponding spacer rings. Similarly, the groove angle cannot be selected arbitrarily due to the availability of parts. The oil level is typically set to standard levels. The dynamic oil viscosity can be set by adjusting the oil temperature. The separator plates can only be planar or waved. Parameters of number of gaps, groove pattern, and clutch sizes were not varied in this study but kept constant. The choice and configuration of the test design are decisive for realizing elementary model properties. The test parameters represent the later model parameters, and the maximum distance between the parameter levels defines the model’s validity range. The modeling of any curvature effects requires investigating at least three parameter levels.

Based on the limitations mentioned above, a mixed-level full factorial design was chosen. The parameters of clearance, dynamic viscosity, and groove angle were investigated in three levels to determine non-linear effects. The shape of the separator plates was investigated in two levels due to the availability of two plate variants. The investigation of the oil level was limited to two levels due to practical relevance. With the selected test design, non-linear effects could also be investigated and mapped in the datasets. Table 3 shows the test designs for Clutch System A and Clutch System B. Each test series consists of 36 measurements. The high and low levels of the parameters represent application-specific values. Additionally, measurements were repeated to validate the test procedure. Adaptive sampling^45^ was also considered, but this approach was not used since certain parameter levels cannot be set arbitrarily due to their categorical nature or the availability of parts. Also, the modification of the clutch set-up is time-consuming. Additionally, screening tests were performed to show differences between dip and injection lubrication. A moderate specific injection flow rate of 0.8 mm^3^/s/mm^2^ (absolute flow rate of 2.4 L/min) was chosen.

**Table 3:** Test designs for Clutch System A and Clutch System B.

Parameter	Low level	Standard level	High level
Clutch System A			
* h*/mm	0.1	–	0.2
* l*/-	hlw	–	lw
* µ*/mPa·s	4.4	13.3	22.1
* ϕ*/°	− 30	0	30
Clutch System B			
* h*/mm	0.1	0.2	0.3
* l*/-	hlw	–	lw
* µ*/mPa·s	11.9	44.2	80.4
Plate shape	Planar	–	Waved

Note: lw, lining width; hlw, half lining width.

**Figure 4:**
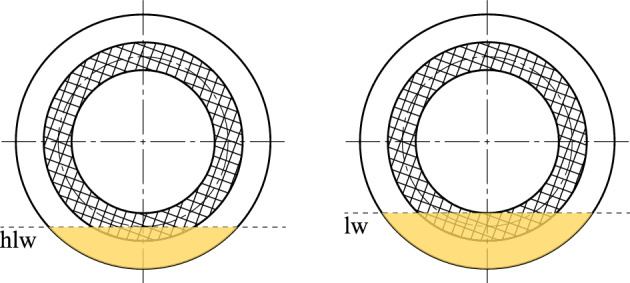
Schematic representation of the investigated oil levels, based on Ref. ^4^. Note: lw, lining width; hlw, half lining width.

The nominal clearance per gap was adjusted via the setting of the total clearance. At oil level lw (lining width), the plates immerse up to the inner diameter, while at oil level hlw (half lining width), the plates only immerse up to the mean diameter (see Fig. 4). The inner carrier does not dip into the oil sump and, therefore, does not cause additional drag losses.

**Figure 5:**
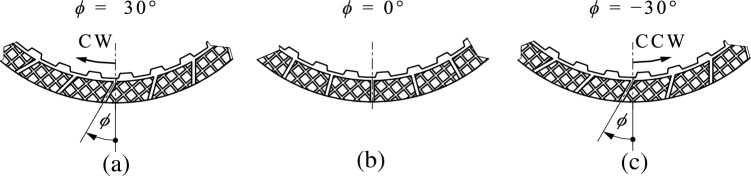
Different groove angles: (**a**) *ϕ* = 30°; (**b**) *ϕ* = 0°; (**c**) *ϕ* =  − 30°. Note: CW, clockwise; CCW, counter-clockwise.

The dynamic viscosity was adjusted via the oil temperature. According to Table 2, the dynamic viscosity at approximately 55 °C represents the center point between the dynamic viscosities at 40 °C and 100 °C oil temperature. The groove angle is referenced to the direction of rotation. In the case of dip lubrication, a conveying behavior results if the grooves are inclined in the direction of rotation (*ϕ* = 30°). Accordingly, a blocking behavior results if the grooves are inclined against the direction of rotation (*ϕ* =  − 30°). Both groove angles were realized by using the same friction plates but reversing the direction of rotation. Figure 5 shows the different groove angles investigated.

### Test evaluation

The tests were evaluated according to the methodology described in Ref.^16^. The mean drag torque was determined for each differential speed step. Transient effects from, for example, the stepwise change in differential speed were excluded from averaging by not considering the first 70% of the step length. Therefore, the mean drag torque was calculated from the last 30% of the step length. By averaging, temperature effects can be compensated. Variations in the oil level were not considered during test evaluation since the variations were to be instantly adjusted by the test rig operator. Compared to the step duration of at least 30 s, correcting the oil level was a very short event. Also, oil level variations were considerably smaller than the difference between the investigated oil levels. To ensure comparability of the results of different clutch sizes and numbers of gaps, the drag torque was related to the mean diameter and the total friction surface according to Eq. 1^27^. This results in the mean shear stress acting within a single gap on the mean diameter.

1 $${\tau }_{{\text{m}}}=\frac{2\cdot {T}_{{\text{d}}}}{{d}_{{\text{m}}}\cdot N\cdot A}$$

**Figure 6:**
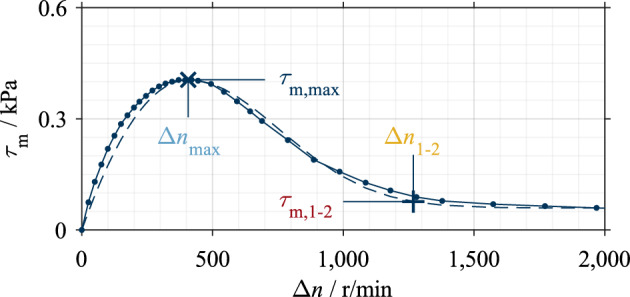
Mean shear stress curve (full line) and its approximation (dashed line). Characteristic drag loss values are marked.

The maximum (coordinates Δ*n*_max_; τ_m,max_) and the transition point from Phase 1 to Phase 2 (coordinates Δ*n*_1−2_; τ_m,1–2_, according to Fig. 1) were determined as characteristic points of the shear stress curve according to Ref.^16^ (see Fig. 6). The four characteristic drag loss values were used for the analysis and represent the support points for the later reconstruction of the shear stress curve as part of the modeling. Figure 6 shows a mean shear stress curve, its approximation, and the characteristic values. The main effects of the influencing parameters on the characteristic values were calculated for systematic analysis. The calculated main effects can further help configure the machine learning algorithm and validate the model.

## Results

**Figure 7:**
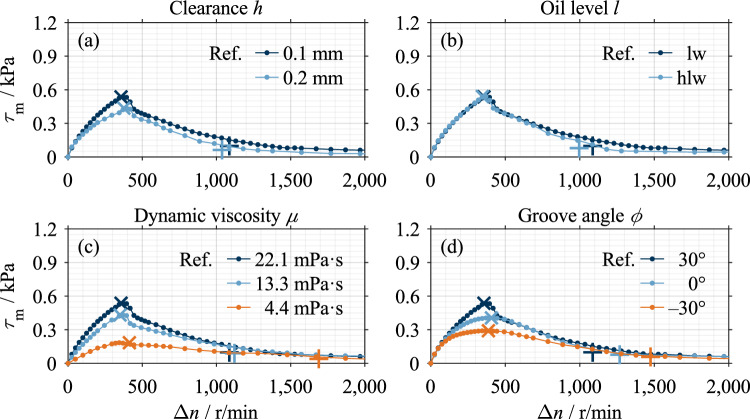
Example measurements Clutch System A: (**a**) Variation of clearance; (**b**) Variation of oil level; (**c**) Variation of dynamic viscosity; (**d**) Variation of groove angle. Note: Reference setting: *h* = 0.1 mm, oil level lw, *µ* = 22.1 mPa·s, *ϕ* = 30°.

**Figure 8:**
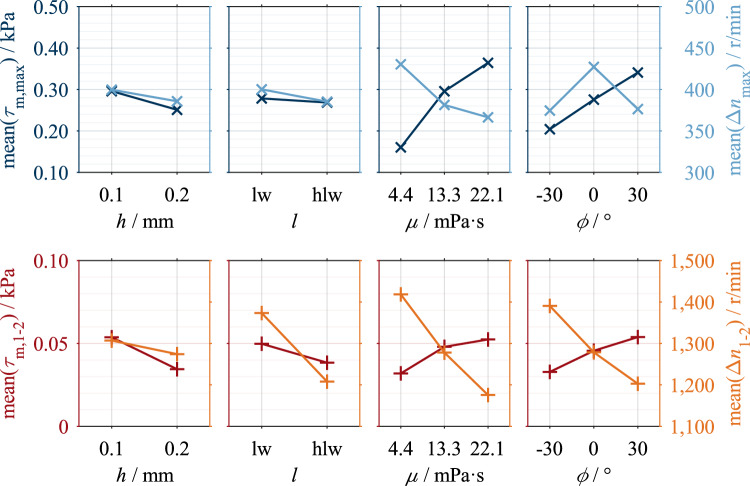
Main effects of investigated parameters on characteristic drag loss values of Clutch System A.

**Figure 9:**
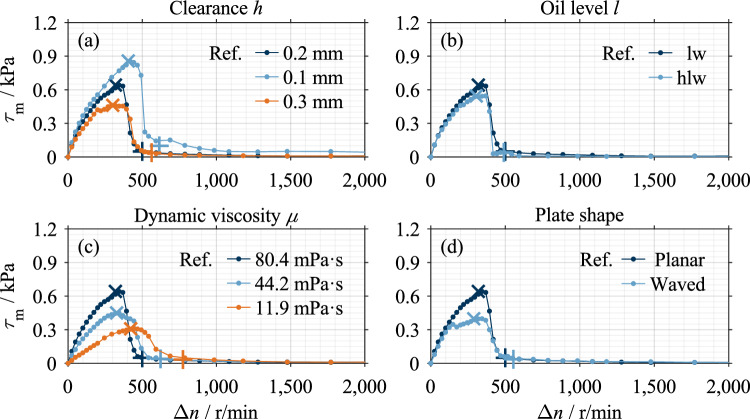
Example measurements Clutch System B: (**a**) Variation of clearance; (**b**) Variation of oil level; (**c**) Variation of dynamic viscosity; (**d**) Variation of plate shape. Note: Reference setting: *h* = 0.2 mm, oil level lw, *µ* = 80.4 mPa·s, planar separator plates.

**Figure 10:**
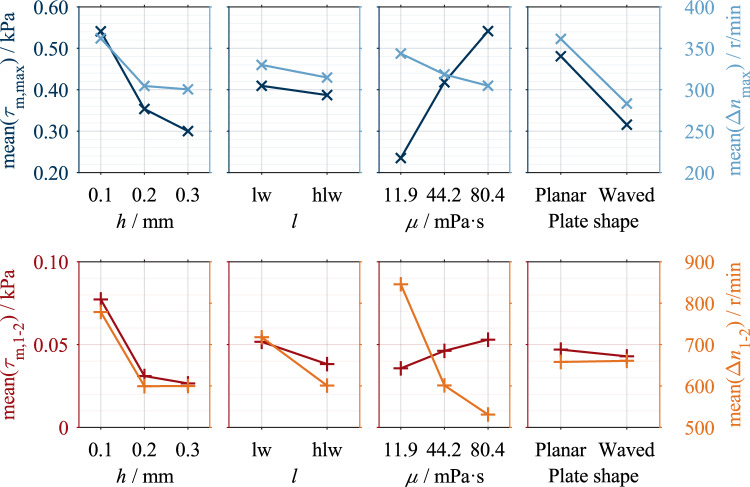
Main effects of investigated parameters on characteristic drag loss values of Clutch System B.

For a better understanding, example results are described for each clutch system. Figure 7 shows the influences of the varied parameters on the drag loss behavior of Clutch System A compared to a reference measurement. Analogously, Fig. 9 visualizes measurements of Clutch System B. The characteristic points of each measurement are marked. The two characteristic points are not necessarily on the curve due to the modeling (see Ref.^16^). All measurements show the characteristic drag loss behavior. Figure 8 shows the main effects of Clutch System A, and Fig. 10 shows the main effects of Clutch System B.

The main effects show that the two characteristic shear stress values, τ_m,max_ and τ_m,1–2_, and the two characteristic differential speed values, Δ*n*_max_ and Δ*n*_1-2_, change in the same trend within a parameter variation. Only the effect of the groove angle on the characteristic differential speed values is an exception. A decrease in the characteristic shear stress values corresponds to a vertical compression of the shear stress curve and, therefore, a reduction in drag losses. A decrease in the characteristic differential speed values corresponds to a horizontal compression of the shear stress curve and, therefore, faster aeration. This can be seen as a reduction in drag losses, too.

The results of the investigations can be seen as datasets. Supplementary Table 1 represents the dataset of Clutch System A, and Supplementary Table 2 represents the dataset of Clutch System B. The datasets contain the influencing parameters investigated as features and the characteristic drag loss values as targets. Each dataset consists of 36 samples. The generated datasets can be used in future research for building data-driven prediction models. The generation of each dataset required approximately 18 h of pure testing time.

### Results of automotive clutch system

The comparison of the curves shows that an increase in clearance, a reduction in dynamic viscosity, and negatively inclined grooves have a significant loss-reducing effect (see Fig. 7 (a,c,d)). At low oil viscosity, the characteristic drop in Phase 1b is comparatively moderate due to the low maximum (see Fig. 7 (c)). Reducing the oil level leads to lower drag losses (see Fig. 7 (b)), although the potential for systematic reduction is limited. In the case of a positive groove angle, the shear stress drops comparatively sharply immediately after the maximum (see Fig. 7 (d)). Nevertheless, the moderate drop in shear stress in Phase 1b is conspicuous.

Figure 8 shows the main effects of the investigated parameters on the characteristic drag loss values. Increasing the nominal clearance and lowering the oil level decreases the characteristic shear stress and differential speed values. An increase in dynamic viscosity increases the characteristic shear stress values but decreases the characteristic differential speed values. The viscosity effect on the characteristic shear stress and differential speed values is non-linear. Increasing the groove angle leads to an increase in the characteristic shear stress values. The effect of the groove angle on the characteristic shear stress values is approximately linear, while there is a non-linear effect for the characteristic differential speed value Δ*n*_max_. The parameters of dynamic viscosity and groove angle show a larger effect size than the parameters of clearance and oil level.

### Results of industrial clutch system

The measurements show that increasing the clearance and reducing the dynamic viscosity has a significant loss-reducing effect (see Fig. 9 (a,c)). Reducing the oil level also leads to lower drag losses (see Fig. 9 (b)), although the potential for systematic reduction is limited. Furthermore, waved separator plates lead to considerably lower drag losses (see Fig. 9 (d)). The shear stress drops sharply in Phase 1b in all measurements.

Figure 10 shows the main effects of the investigated parameters on the characteristic drag loss values. Increasing the nominal clearance and lowering the oil level decreases the characteristic shear stress and differential speed values. The effect of clearance on the characteristic shear stress and differential speed values is non-linear. An increase in dynamic viscosity results in an increase in the characteristic shear stress values but a decrease in the characteristic differential speed values. The effect of oil viscosity on both the characteristic shear stress and differential speed values is non-linear. The use of waved separator plates leads to a decrease in the characteristic shear stress and differential speed values. The parameter of oil level shows a minor effect size compared to the parameters of clearance, dynamic viscosity, and plate shape.

### Repeatability

**Figure 11:**
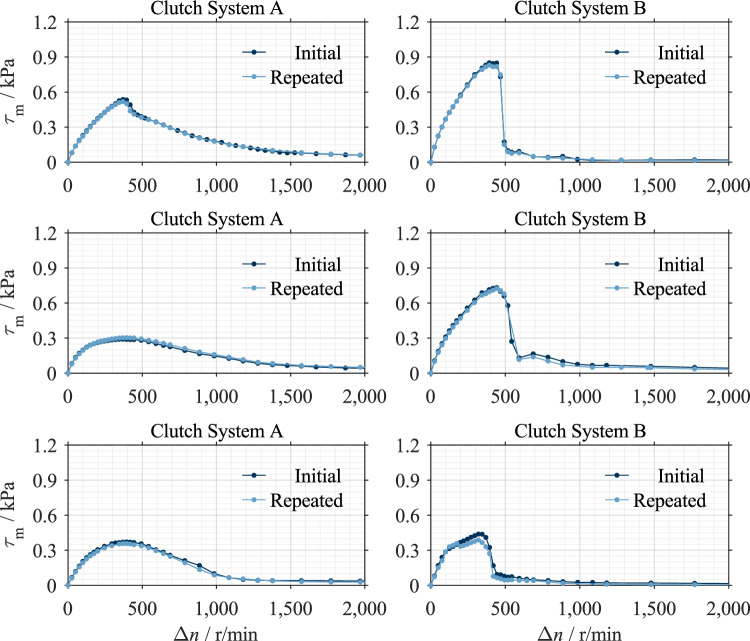
Proof of repeatability of the measurements.

Measurements were repeated to prove repeatability. The clutch was dismounted and remounted between the initial and repeated measurements. Figure 11 shows that the measurements nearly overlap throughout the differential speed range.

## Discussion

The following interpretations and findings are based on the low relative uncertainty of the drag torque measurement of approx. 1% in the relevant drag torque range (see Supplementary Fig. 5). The preliminary study^7^ used a two-level full factorial design to systematically investigate the drag loss behavior. No findings on curvature effects have been generated with this test design.

### Dip lubrication versus injection lubrication

The influences of various parameters on drag loss behavior have already been extensively investigated for injection lubrication. Although the flow in the gaps develops differently between injection^23^ and dip lubrication^4^, the results show comparable integral effects of various influencing parameters for both types of lubrication. During dip lubrication, air enters the gaps from the inside, beginning at a low differential speed^4^. The oil is then continuously displaced from the gaps due to the increasing centrifugal force^4^. Supplementary Fig. 6 exemplarily shows the flow development during dip lubrication. In contrast, during injection lubrication, air enters the gaps from the outside^23^ when the conveying rate of the clutch exceeds the flow rate supplied. Independent of the type of lubrication and under typical operating conditions, the gaps are initially filled with oil, while a mix of oil and air is present at higher differential speeds^4,23^. The fraction of air increases with increasing differential speed^4,24^. Thus, both types of lubrication lead to a comparable drag loss behavior. The integral effects of various parameters on the drag loss behavior can, therefore, be interpreted identically for both types of lubrication.

**Figure 12:**
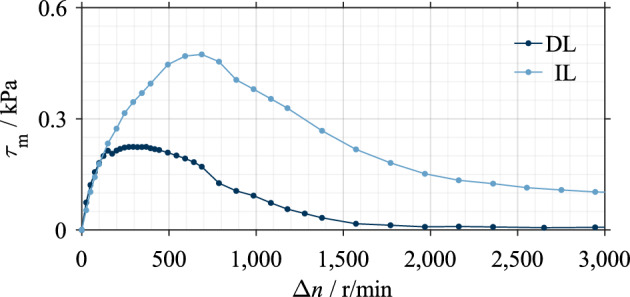
Comparison of dip and injection lubrication based on Clutch System A. Note: Settings: *h* = 0.2 mm, *µ* = 22.1 mPa·s, *ϕ* = 30°, oil level lw during dip lubrication (DL), specific injection flow rate 0.8 mm^3^/s/mm^2^ during injection lubrication (IL).

Figure 12 compares the drag loss behavior of dip and injection lubrication based on Clutch System A. In the case of injection lubrication, the single-phase flow can be maintained up to higher differential speeds due to the active oil supply. During dip lubrication, the gaps are oil-free at high differential speed, resulting in nearly no drag losses. In contrast, the drag losses remain remarkably at a high differential speed due to the continuous oil supply during injection lubrication.

All measurements performed show the characteristic drag loss behavior. However, the difference in the shape of Phase 1b between the clutch systems (see Fig. 7 and Fig. 9) is conspicuous.

The generation of drag loss can be explained using Newton’s law of viscosity. According to Newton’s law of viscosity, the shear stress is proportional to the dynamic viscosity and the shear rate. An incompressible fluid and a laminar flow are assumed. In the idealized case of a Couette flow, the shear rate is the ratio of the plates’ differential velocity and the clearance. The drag torque is proportional to the area of the wetted surface. The drag torque of a single fully-filled gap (*r*_ib_ = *r*_i_) can be calculated using Eq. 2^20^, assuming a single-phase flow. The grooving of the friction plates is not considered.

2 $$T_{{\text{d}}} \left( {{\Delta }n} \right) = \frac{{\pi^{2} \cdot \mu }}{h} \cdot {\Delta }n \cdot \left( {r_{{\text{o}}}^{4} - r_{{{\text{ib}}}}^{4} } \right)$$

**Figure 13:**
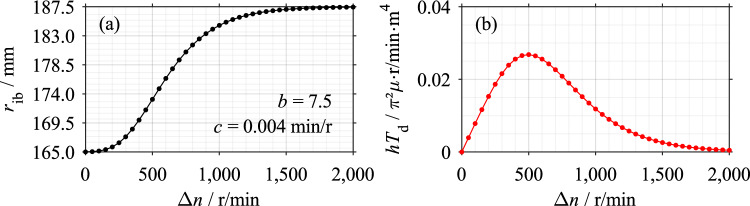
Modeling of the continuous oil displacement from the gaps based on Clutch System A: (**a**) Inner boundary of oil ring; (**b**) Resulting drag loss curve.

To adapt Eq. 2 for dip lubrication, the inner boundary of the oil ring *r*_ib_(Δ*n*) is modeled speed-dependent to consider the continuously shrinking of the oil ring (see Supplementary Fig. 6). Based on Eq. 2, the characteristic drag loss curve results if the continuous oil displacement is modeled by a sigmoid function as per Eq. 3 (see Fig. 13).

3 $${r}_{{{\text{ib}}}}(\Delta n)={r}_{{\text{i}}}+({r}_{{\text{o}}}-{r}_{{\text{i}}})\cdot {e}^{{-b}\cdot {e}^{-c\cdot\Delta n}}$$

The sigmoid function depicts the initial fully-filled state and the subsequent continuous displacement (see Fig. 13 (a)) as described in Ref.^4^ and as shown in Supplementary Fig. 6. As the differential speed increases, the shear stress increases due to the increasing shear rate, while at the same time, the oil-wetted area decreases. The characteristic drag loss behavior is a superposition of both effects (see Fig. 13 (b)). The drag torque resulting from the shearing of the air in the oil-free region is neglected.

The form of the oil displacement, therefore, determines the characteristic shape of the drag torque curve. Thus, the different shapes of the shear stress curves of the two clutch systems can be traced back to the displacement process. Based on this finding and the different shear stress curves in Figs. 7 and 9, it can be concluded that the displacement process is, in turn, influenced by the geometry and operating parameters. The test set-up used in this study only allows for the determination of integral effects. A detailed analytical or simulative study should be conducted to validate the described explanatory approach.

### Effects of influencing parameters on drag loss behavior

The effects of dynamic viscosity and clearance (see Figs. 8 and 10) can be explained using Eq. 2. According to Eq. 2, the drag torque increases directly proportional to the dynamic viscosity and decreases with inverse proportionality to the clearance. For Clutch System B, the inversely proportional decrease in shear stress with increased clearance was found (see Fig. 10). However, the linear relationship between dynamic viscosity and shear stress can only be observed for Clutch System B (see Fig. 10). Clutch System A, in contrast, shows a degressive behavior (see Fig. 8). Further investigations are required to explain this behavior. In the preliminary study^7^, similar trends of both parameters were determined but only investigated in two levels. The general effects determined for the parameters of clearance and viscosity were also found for injection lubrication^3,18–20^.

With a higher oil level, a higher centrifugal force is generally required to displace the oil from the gaps^4^. Consequently, lowering the oil level leads to lower characteristic shear stress and differential speed values (see Figs. 8 and 10). However, the effects of the oil level are of minor size. This can be traced back mainly to the slight difference between the investigated levels. In the case of Clutch System A, the difference between the oil levels is 5.6 mm, whereas in the case of Clutch System B, it is 11.3 mm. A recent study^4^ showed that the flow in the gaps develops almost identically for the two oil levels investigated. The studies^7,18,27^ found similar effects of the oil level on the drag losses.

The effects of the groove angle (see Fig. 8) can be traced back to the different flow development in the gaps. Due to the delayed oil displacement in the case of a positive groove angle, higher characteristic shear stress values result. The non-linear behavior of the characteristic differential speed value Δ*n*_max_ can be traced back to the peak-like maximum caused by the sudden drop in shear stress in the case of a positive groove angle (see Fig. 7 (d)). In the case of a negative groove angle, the maximum is plateau-like, which results in higher values of the characteristic differential speed value Δ*n*_max_. In contrast, the characteristic differential speed value Δ*n*_1-2_ shows a linear effect. However, further investigations are required to explain the sudden drop in shear stress. The effect of the groove angle has also been found for injection lubrication^15,20,22,30^. In contrast, a negative groove angle causes a conveying effect due to the oil supply from the inside^20^.

In the case of waved separator plates, the clearance is the sum of the set nominal clearance and the waviness of the plate, meaning the circumferential clearance varies sinusoidally. Thus, the mean clearance is larger than in the case of planar plates. Therefore, the integral effects determined (see Fig. 10) can be interpreted as an increase in clearance. The loss-reducing effect of waved plates can also be seen during injection lubrication^3,17,18^.

However, during the development process, design trade-offs must be considered. Increasing the clearance, e.g., simultaneously reduces the clutch reaction time in operation and increases the likelihood of torque jump^26^. Further, reducing the plate number, and, thus, the number of gaps reduces the transmittable torque, whereas decreasing the oil viscosity affects the torque response during engagement^46^. In the case of injection lubrication, decreasing the flow rate, e.g., may negatively affects the cooling performance resulting in damage of the clutch system in the worst case^47^. Hence, optimizing the drag loss behavior has to be done under consideration of the friction and thermal behavior.

### Dataset use for data-driven modeling

Generally, the datasets determine elementary model properties such as the dimension or the validity range. Due to the test designs underlying the datasets, four-dimensional prediction models can be built. For model building, Gaussian process regression and symbolic regression are recommended^16^. The gained knowledge about the effects of the individual parameters can be used to choose and configure the machine learning algorithm and validate the trained models. When using Gaussian process regression, the findings on the curvature effects can be used for the selection and specification of the kernel functions. In the case of symbolic regression, the findings can be used for initializing the set of potential mathematical functions.

**Figure 14:**
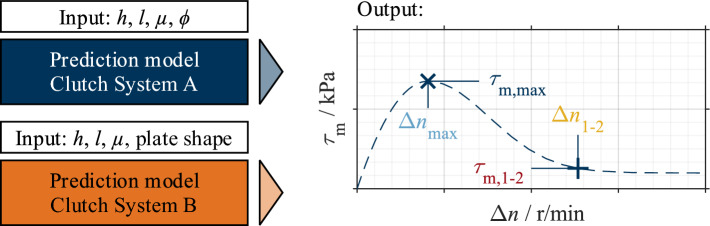
Input and output of prediction models.

The predicted values serve as support points for reconstructing the shear stress curve. The definition of the approximation function is described in detail in Ref.^16^. Figure 14 shows an example of the input and output of the models that can be built with the generated datasets.

## Conclusion

This paper describes new findings on the drag loss behavior of dip-lubricated wet clutches. The investigations were designed and conducted so that the generated research data and findings can be used in further research for building data-driven drag loss prediction models.

The form of oil displacement from the gaps mainly determines the characteristic drag loss behavior. The displacement process is influenced by the operating and geometry parameters. In the case of dip lubrication, drag losses can be significantly reduced by increasing the clearance, reducing the oil viscosity, and choosing waved plates. Reducing the oil level also leads to lower drag losses. Nevertheless, the potential for systematic reduction is limited.

The generated datasets and gained knowledge can be used in further research for building data-driven prediction models. The prediction models can be used for designing new low-loss clutch systems and as part of full powertrain simulation models. This paper represents a template for the generation and analysis of datasets for data-driven modeling of drag losses of wet clutches.

### Supplementary Information

Supplementary Information.

## Data Availability

The datasets generated or analyzed during the current study are available as supplementary material.

## List of symbols

*A*: Friction area per interface (mm^2^)
*b*: Coefficient sigmoid function (-)
*c*: Coefficient sigmoid function (min/r)
*d*: Diameter (mm)
*h*: Clearance (mm)
*l*: Oil level (mm)
*n*: Speed (r/min)
*N*: Number of gaps (-)
*r*: Radius (mm)
*t*: Time (s)
*t*_CP_: Thickness of clutch pack (mm)
*T*_d_: Drag torque (Nm)
*w*_l_: Lining width (mm)
Δ*n*: Differential speed (r/min)
*µ*: Dynamic viscosity (mPa·s)
*ν*: Kinematic viscosity (mm^2^/s)
*ρ*: Density (kg/m^3^)
*τ*: Shear stress (kPa)
*ϕ*: Groove angle (°)

## Indices

1–2: Transition Phase 1–Phase 2
i: Inner
ib: Inner boundary of oil ring
m: Mean
max: Maximum
o: Outer
