# Supplementary material for: Experimental investigation of drag loss behavior of dip-lubricated wet clutches for building a data-driven prediction model
Source: Sci Rep. 2024 Apr 22;14:9241. doi: 10.1038/s41598-024-59488-4 (PMC11035595; doi:10.1038/s41598-024-59488-4)
Supplement: Supplementary file 1 — Supplementary Information. [file 41598_2024_59488_MOESM1_ESM.pdf]

Experimental investigation of drag loss behavior of dip-lubricated wet clutches for building a data-driven prediction model

Lukas Pointner-Gabriel, Max Menzel, Katharina Voelkel, Thomas Schneider, Karsten Stahl

Technical University of Munich, School of Engineering and Design, Department of Mechanical Engineering, Gear Research Center (FZG), 85748 Garching near Munich, Germany

Data-driven modeling and prediction

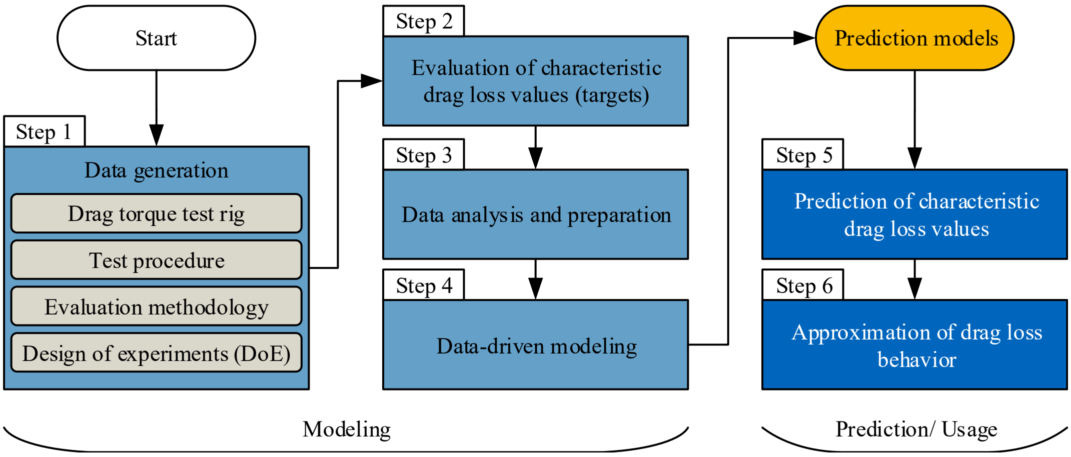

Supplementary Figure 1: Methodology for data-driven modeling and prediction of drag losses of wet clutches, based on Ref. [16].

Graphical display of clutch systems

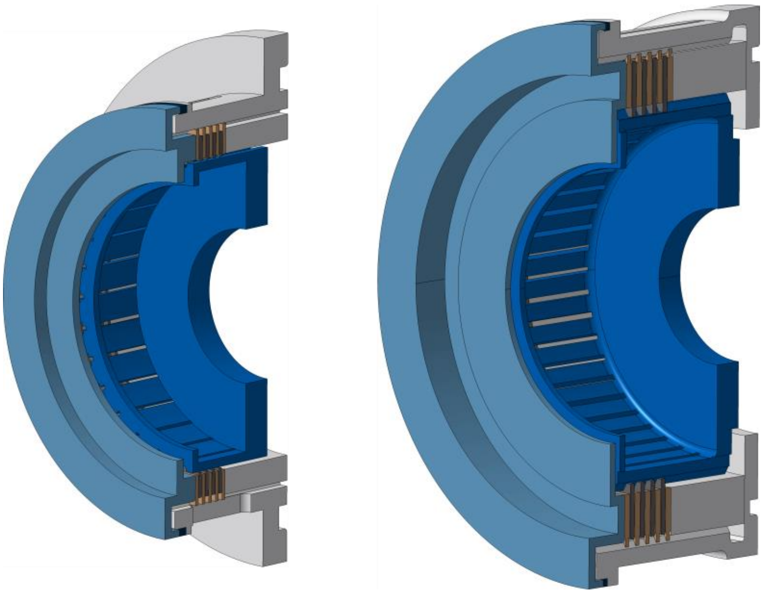

Supplementary Figure 2: Graphical display of Clutch Systems A (left) and B (right) in sectioned view in test rig configuration.

LK-4 drag torque test rig

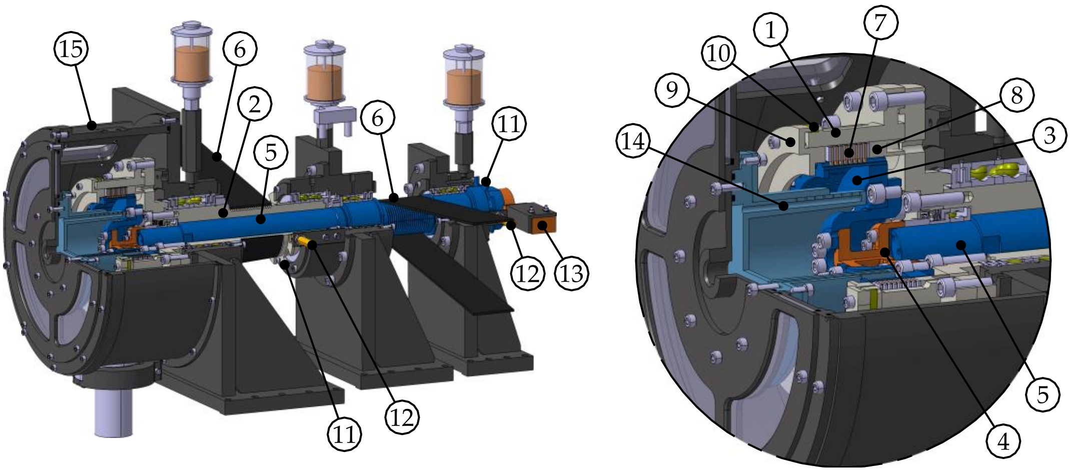

(1) Outer carrier	(6) V-ribbed belt	(11) Inductive incremental encoder
(2) Hollow shaft	(7) Clutch pack	(12) Inductive incremental encoder
(3) Inner carrier	(8) Spacer ring	(13) Telemetry system
(4) Measuring shaft	(9) Closing cover	(14) Oil nozzle
(5) Full shaft	(10) Spacer ring	(15) Housing

Supplementary Figure 3: Graphical display (isometric view (left) and detail (right)) of the LK-4 drag torque test rig [16].

Set-up for determination of clutch pack thickness

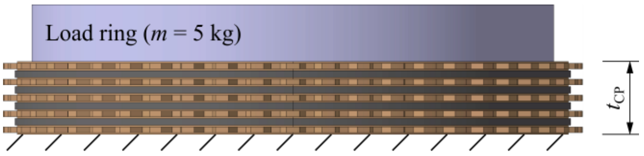

Supplementary Figure 4: Set-up for determination of the clutch pack thickness t_{CP}.

Measurement uncertainty

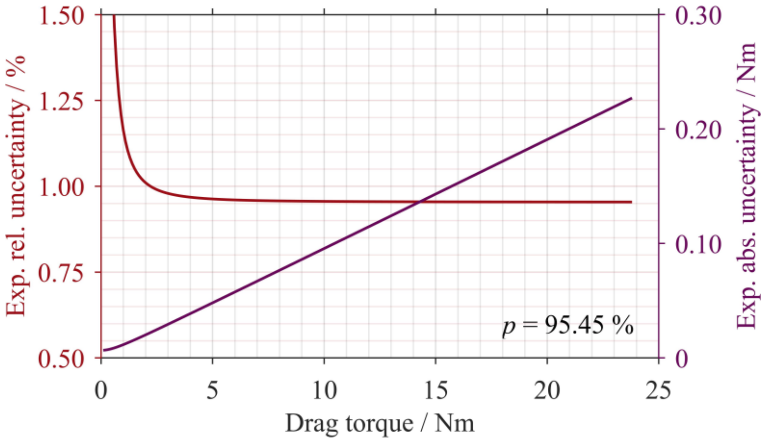

Supplementary Figure 5: Expected relative and absolute measurement uncertainty of the drag torque calculated using the GUM method [4].

Flow development during dip lubrication

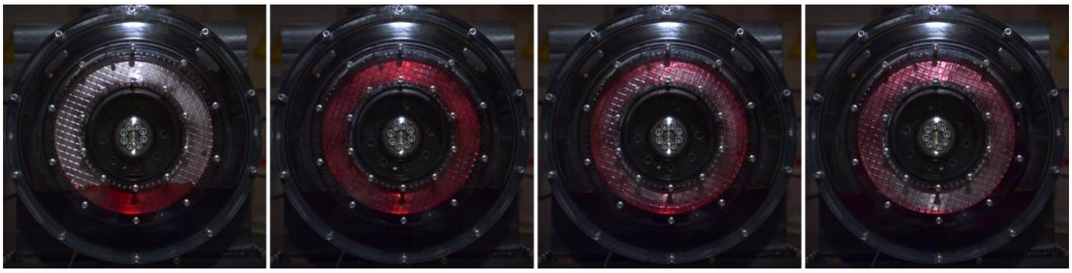

(a) Standstill (b) Beginning of Phase 1a (c) Beginning of Phase 1b (d) Beginning of Phase 2

Supplementary Figure 6: Flow development during dip lubrication for characteristic differential speeds, based on Ref. [4].

Generated datasets

Supplementary Table 1: Features and targets of Dataset A (Clutch System A).

A	Features				Targets			
	h	l	μ	ϕ	$\tau_{m,max}$	Δn_{max}	$\tau_{m,1\text{-}2}$	$\Delta n_{1\text{-}2}$
Sample	mm	-	mPa·s	°	kPa	r/min	kPa	r/min
1	0.1	'lw'	22.1	0	0.380	414.71	0.064	1264.63
2	0.1	'hlw'	22.1	0	0.364	395.34	0.053	1187.99
3	0.1	'lw'	13.3	0	0.314	425.70	0.061	1341.37
4	0.1	'hlw'	13.3	0	0.304	419.16	0.049	1234.00
5	0.1	'lw'	4.4	0	0.174	486.63	0.041	1467.32
6	0.1	'hlw'	4.4	0	0.171	466.98	0.034	1313.42
7	0.2	'lw'	22.1	0	0.355	375.75	0.055	1186.46
8	0.2	'hlw'	22.1	0	0.343	374.29	0.043	1128.80
9	0.2	'lw'	13.3	0	0.289	418.21	0.048	1301.92
10	0.2	'hlw'	13.3	0	0.280	413.58	0.041	1228.26
11	0.2	'lw'	4.4	0	0.167	472.56	0.032	1408.14
12	0.2	'hlw'	4.4	0	0.164	463.96	0.025	1279.80
13	0.1	'lw'	22.1	30	0.538	353.58	0.096	1060.00
14	0.1	'hlw'	22.1	30	0.540	346.08	0.079	995.79
15	0.1	'lw'	22.1	-30	0.294	391.37	0.056	1470.24
16	0.1	'hlw'	22.1	-30	0.283	372.18	0.042	1156.08
17	0.1	'lw'	13.3	30	0.432	350.22	0.090	1120.89
18	0.1	'hlw'	13.3	30	0.393	335.89	0.057	1138.26
19	0.1	'lw'	13.3	-30	0.247	402.73	0.052	1625.24
20	0.1	'hlw'	13.3	-30	0.245	394.72	0.051	1235.06
21	0.1	'lw'	4.4	30	0.184	413.20	0.040	1690.00
22	0.1	'hlw'	4.4	30	0.176	385.93	0.035	1392.40
23	0.1	'lw'	4.4	-30	0.147	432.86	0.036	1645.57
24	0.1	'hlw'	4.4	-30	0.144	407.38	0.030	1185.75
25	0.2	'lw'	22.1	30	0.435	376.02	0.063	1037.65
26	0.2	'hlw'	22.1	30	0.397	355.22	0.041	984.31
27	0.2	'lw'	22.1	-30	0.224	346.25	0.022	1447.92
28	0.2	'hlw'	22.1	-30	0.217	296.47	0.016	1186.25
29	0.2	'lw'	13.3	30	0.328	362.50	0.047	1140.23
30	0.2	'hlw'	13.3	30	0.329	354.00	0.032	1133.68
31	0.2	'lw'	13.3	-30	0.193	338.57	0.026	1506.46
32	0.2	'hlw'	13.3	-30	0.195	361.51	0.020	1329.41

33	0.2	'lw'	4.4	30	0.180	458.99	0.044	1438.22
34	0.2	'hlw'	4.4	30	0.158	425.20	0.022	1300.72
35	0.2	'lw'	4.4	-30	0.130	382.88	0.023	1566.91
36	0.2	'hlw'	4.4	-30	0.131	368.09	0.021	1330.77

Supplementary Table 2: Features and targets of Dataset B (Clutch System B).

B	Features				Targets			
	h	l	μ	Plate shape	$\tau_{m,max}$	Δn_{max}	$\tau_{m,1\text{-}2}$	$\Delta n_{1\text{-}2}$
Sample	mm	-	mPa·s	-	kPa	r/min	kPa	r/min
1	0.1	'lw'	80.4	'Planar'	0.905	398.85	0.098	598.99
2	0.1	'hlw'	80.4	'Planar'	0.840	380.12	0.073	580.15
3	0.1	'lw'	44.2	'Planar'	0.728	406.26	0.088	686.37
4	0.1	'hlw'	44.2	'Planar'	0.722	391.44	0.073	644.08
5	0.1	'lw'	11.9	'Planar'	0.447	458.80	0.079	871.04
6	0.1	'hlw'	11.9	'Planar'	0.424	439.67	0.056	801.47
7	0.2	'lw'	80.4	'Planar'	0.615	310.10	0.048	511.14
8	0.2	'hlw'	80.4	'Planar'	0.543	299.92	0.039	482.57
9	0.2	'lw'	44.2	'Planar'	0.449	327.12	0.040	623.20
10	0.2	'hlw'	44.2	'Planar'	0.432	328.55	0.032	594.04
11	0.2	'lw'	11.9	'Planar'	0.308	421.87	0.034	774.27
12	0.2	'hlw'	11.9	'Planar'	0.264	392.60	0.024	822.75
13	0.3	'lw'	80.4	'Planar'	0.461	301.09	0.038	563.22
14	0.3	'hlw'	80.4	'Planar'	0.422	261.19	0.029	443.34
15	0.3	'lw'	44.2	'Planar'	0.340	302.26	0.028	611.08
16	0.3	'hlw'	44.2	'Planar'	0.308	295.04	0.024	582.78
17	0.3	'lw'	11.9	'Planar'	0.209	388.43	0.019	866.72
18	0.3	'hlw'	11.9	'Planar'	0.237	401.60	0.021	787.88
19	0.2	'lw'	80.4	'Waved'	0.408	260.80	0.035	553.22
20	0.2	'hlw'	80.4	'Waved'	0.394	264.93	0.028	482.77
21	0.2	'lw'	44.2	'Waved'	0.294	268.84	0.030	618.81
22	0.2	'hlw'	44.2	'Waved'	0.269	256.29	0.019	558.06
23	0.2	'lw'	11.9	'Waved'	0.134	269.07	0.027	596.53
24	0.2	'hlw'	11.9	'Waved'	0.131	254.28	0.015	576.59
25	0.3	'lw'	80.4	'Waved'	0.413	281.66	0.043	534.01
26	0.3	'hlw'	80.4	'Waved'	0.345	249.86	0.025	466.42
27	0.3	'lw'	44.2	'Waved'	0.296	310.77	0.030	554.49
28	0.3	'hlw'	44.2	'Waved'	0.292	289.53	0.021	512.75
29	0.3	'lw'	11.9	'Waved'	0.140	270.80	0.024	711.42
30	0.3	'hlw'	11.9	'Waved'	0.137	253.47	0.016	565.01
31	0.1	'lw'	80.4	'Waved'	0.578	334.28	0.102	614.85
32	0.1	'hlw'	80.4	'Waved'	0.575	315.05	0.076	541.58
33	0.1	'lw'	44.2	'Waved'	0.447	333.70	0.102	653.01
34	0.1	'hlw'	44.2	'Waved'	0.439	312.50	0.067	578.32
35	0.1	'lw'	11.9	'Waved'	0.197	294.77	0.064	1977.61
36	0.1	'hlw'	11.9	'Waved'	0.191	278.95	0.047	795.89
